# Retention and Clinical Performance of Zirconia Crowns: A Comprehensive Review

**DOI:** 10.1155/2020/8846534

**Published:** 2020-10-15

**Authors:** Fatemeh Soleimani, Hamid Jalali, Azam Sadat Mostafavi, Somayeh Zeighami, Maryam Memarian

**Affiliations:** Department of Prosthodontics, School of Dentistry, Tehran University of Medial Sciences, Tehran, Iran

## Abstract

Zirconia has been used for rehabilitation of edentulous spaces approximately for a decade, and there have been several reports regarding the clinical performance and retention of zirconia crowns. Outstanding mechanical properties, biocompatibility, and excellent aesthetics make zirconia-based crowns as a popular crown among the current all-ceramic crowns in restorative dentistry. However, restoration with a zirconia crown is a challenging treatment. The goal of this study was to assess the current literature to summarize the studies reporting the effective risk factors on retention of zirconia crowns to provide clinicians with a useful point of view in the decision-making process for use of these restorations. Literature based-search was performed to find related articles until August 2020 using EMBASE, Google Scholar, and MEDLINE. Search terms used were “zirconia restorations properties,” “zirconia crowns clinical performance,” “zirconia crown survival,” “biological complications,” and “zirconia crown retention.” Results were limited to papers available in English. The references of all related literature were also searched for further citations. Overall, although clinical long-term and follow-up studies are a vital requirement to conclude that zirconia has great reliability, it seems that zirconia crown restorations are both well tolerated and sufficiently resistant.

## 1. Introduction

The treatment of edentulous spaces with an osseointegrated dental implant is a known treatment modality and scientifically accepted [1]. Every implant complex differs from the others for many features including materials, implant shape, roughness, spirals, geometry, and connection level [2]. Over years ago, research studies were performed on all these various characteristics to finding the most efficient, valuable, and safe implant complex in short- and long-term follow-up [2]. One of the most challenging aspects of edentulous spaces rehabilitation is to satisfy the patient with an acceptable outcome of the clinical performance of various parts in a dental implant.

During the last few decades, because of esthetic quality and high success rate, metal-ceramic crowns have been the restoration of choice [3]. Recently, Zirconia has been tested showing excellent hardness [4], marginal fit [5], and bond strength [6]. Nevertheless, restoration with a zirconia crown is a challenging treatment, and clinical problems related to the zirconia crown may decrease the retention form and clinical performance of this restoration [7].

Despite the probable problems, recently, the demand for zirconia, due to the zirconia mechanical properties, is increasing considerably. Since zirconia-based restorations are a topic of current interest in the light of recent studies that have demonstrated the excellent biological and mechanical properties of these restorations, a comprehensive review introducing effective factors on its retention is warranted. The goal of this study was to assess the current literature to summarize the studies reporting the effective risk factors on retention of zirconia restorations in crowns to provide clinicians with a useful point of view in the decision-making process for use of these restorations. Literature based-search was performed to find related articles until August 2020 using EMBASE, Google Scholar, and MEDLINE. Search terms used were “zirconia restorations properties,” “zirconia crowns clinical performance,” “zirconia crown survival,” and “zirconia crown retention.” Results were limited to papers available in English. The references of all related literature were also searched for further citations. We assessed subjects, abstracts, and the full-text paper of the records retrieved from the databases for probable inclusion. Data obtained are categorized as follows.

## 2. Mechanical Properties

Zirconia (zirconium dioxide, ZrO_2_) has some features (low corrosion potential, low thermal conductivity, good biologic compatibility, and good radiographic contrast) making it the material of choice where high functional and esthetic issues are concerned [8]. The mechanical properties of zirconia are the highest ever reported for any dental ceramic, which naturally has generated extensive interest [9]. Zirconia is a polymorphic crystal that is categorized into three crystallographic forms: tetragonal, cubic, and monoclinic. At room temperature, zirconia is monoclinic and stable till 1170°C. Over this temperature, it becomes tetragonal, and above 2370°C temperature, it passes to the cubic phase [10]. Most of the zirconia-based ceramic complexes that are, at present, used in the rehabilitation of edentulous spaces are yttrium-stabilized zirconia polycrystals (3Y-TZP) [9]. This zirconia contains 2% to 3% mol of yttria (Y_2_O_3_) as a stabilizer. Compared with other all-ceramic core materials, the most important advantage of Y_2_O_3_ is their high fracture resistance which represents by their superior flexural strength and fracture toughness [11].

The mechanical properties of zirconia depend upon characteristics including composition, nature of crystals and finish line on the implant abutment, ratio of the monoclinic to tetragonal phase, metastable polymorphic structure, percentage of Y_2_O_3_ stabilizer, the aging process, type of implant design, and amount of occlusal load [12].

### 2.1. Transformation

Although transformation tighten increases the fracture strength and toughness of Y-TZP implant, it prevents the phase integrity and makes the zirconia restorations susceptible to low-temperature degradation (LTD) and facilitate the aging process. A raise in stress or moisture on zirconia may cause the transformation of its crystals to a monoclinic phase with the formation of microcracks that raise water penetration, surface deterioration, and crack propagation, resulting in decreased resistance to overload [13]. It has been shown that metastability, mechanical properties, and resistance to LTD of zirconia are influenced by the porosity of the material, stabilizer content, amount of cubic phase, and dental procedures such as sandblasting or grinding [14]. Even though no clinical evidence of LTD has yet been shown for zirconia restorations, this slow but autocatalytic phenomenon potentially is triggered by a combination of high sintering temperatures, lower-grade powders, and direct exposure to oral fluids [13, 15].

### 2.2. Fracture Resistance

Fracture resistance of zirconia is strongly affected by the amount of occlusal load and characteristics of the implant-abutment [16]. When a heavy occlusal load is exerted on the zirconia-based crowns, fracture of the implant-abutment is more for one-piece zirconia restorations under unloaded than those under loaded conditions [17–19]. Kohal et al. performed a pilot in vitro study to compare the mechanical properties and effect of occlusal load on zirconia and revealed that the fracture strength was lower for two-piece zirconia in loaded and unloaded conditions [20]. Kohal et al. also assessed the effects of finish line design and cyclic loading on the fracture strength of zirconia and shown that chamfer finish lines along with cyclic loading reduce the fracture strength in zirconia restorations [21]. In 2003, Yildirim et al. [22] investigated the fracture resistance of zirconia and alumina under static loads. They concluded that zirconia obtained fracture resistance values more than twice higher than alumina. In addition, it has shown that the nature and type of preparation also influenced the fracture resistance of zirconia. The current preparation procedure for zirconia crowns does not recommend deeper preparation than that for metal-ceramic crowns because the core can be made only 0.5 mm thick. However, the relatively high rate of loss of retention in zirconia crowns could possibly be related to cement used or tooth preparation. Silva et al. carried out an in vitro study in order to examine the effects of preparation on zirconia crowns and clarified that the fracture strength with full preparation was higher compared to that without preparation [19].

### 2.3. Optical Behavior and Biocompatibility

A main advantage of zirconia compared with titanium is attributed to its outstanding aesthetics. The optical behavior of zirconia differs with its composition, crystal size, and machining methods applied [23]. The improved aesthetics of zirconia is in relation to its capability to mask dark substrates with excellent opacity in the infrared and visible spectrum. The masking capability is because of its grain size being greater than the light length, low absorption coefficient, high refractive index, high density with low residual porosity (<0.05%), and the presence of different additives and stabilizers [24–26]. The biocompatibility properties of the zirconia are even enhanced than titanium ones. The bacterial adhesion, which is essential in the maintaining of zirconia, was demonstrated acceptably low [27, 28]. Scarano et al. [28] reported a low degree of bacterial adhesion in the zirconia (12.1%) in comparison with titanium (19.3%). An in vivo study performed by Rimondini et al. [27] demonstrated Scarano et al.'s study results.

Considering the type of material used in restorations, it has shown that zirconium oxide induces a lesser phlogistic reaction than titanium restorative materials [29]. In addition, inflammatory infiltrate, vascular endothelial growth factor expression, and microvessel density were reported to be lower around the zirconia restorations than around the titanium ones [30]. Zirconia can affect the expression levels of some genes in such a way that zirconia can be known as a self-regulatory material that can affect turnover of the extracellular matrix [31]. In addition, Rinke et al. showed that endodontic failures as a biological complication result in a reduced survival rate of approximately 74% in zirconia crowns after the 7-year follow-up study [32]. The recent finding is consistent with other clinical trials reporting on zirconia crowns [33, 34], which have reported reduced survival rates for this material on endodontically treated teeth.

## 3. Retention and Clinical Performance

Given the increasing popularity of zirconia, several complications have reported in the literature. Various researchers have reported the causes of zirconia restorations failure. According to the studies of layered zirconia, chipping of the veneering porcelain is the most commonly reported complication [35–37]. Besides inherent defects as cause of ceramic failure, zones of occlusal contact wear have been known as primary starting points of chipping fracture [38]. Posterior crowns were described to be principally susceptible to chipping [39]. Residual thermal stresses, a mismatch of the coefficient of thermal expansion, and differences in the moduli of elasticity between the zirconia and the veneering material can be other contributing factors in chipping fracture. To overcome these complications, the overpressing technique has been introduced. In this technique, a specific ceramic is pressed onto the zirconia framework [40]. Beuer et al. [41] confirmed that the overpressing technique for veneering porcelain led to no chipping, and it seems that this technique can be reliable.

Some authors suggested that an ideal luting agent provides sufficient retention for ceramic crowns. In general, luting cements contain at least two mixed pastes. It has been shown that voids and air bubbles generated in mixed pastes may introduce into the cementation agent and lead to degradation of mechanical features [42]. Clinical data reveal that the type of luting agent used can affect the retention of zirconia-based crowns, and the durability of an implant is dependent on the type and quality of luting agents [43]. However, obtaining useful information on the effect of type and quality of luting agent needs further research. Studies have also shown that air abrasion affected retention not considering the cement type used [44, 45]. By generating microcracks which can minimize the fracture strength of the ceramic, air abrasion can influence the ceramic surface [46]. Some studies indicated that the machining process generates a surface roughness [44, 47], and this surface roughness enhances the micromechanical interlocking of luting agents to the surface of ceramic [48]. It is also worth mentioning that some authors showed increased bond strength to zirconia ceramic after air abrasion [49–51]. Wolfart et al. [49] carried out an in vitro study and indicated that air abrasion in zircon ceramic luted with various cements produces the highest tensile bond strength in comparison with other surface-conditioning methods.

Cement thickness is another main parameter influencing the retention of the crown. Nevertheless, when cement space is insufficient, it will cause crown seating discrepancies. The cement space can improve the outflow of excess cement and seating of a crown, lower the seating forces, and decrease the elevation of restorations, consequently resulting in retention of the restoration [52, 53]. The data of Gultekin et al.'s study showed that, for the lower strength cements, increasing the cement space from 20 to 40 *μ*m did not have any considerable effect on restoration retention [52]. The finding of Gultekin et al.'s study agreed with the finding of another study by Alaa Abou-Obaid. These results agree with other studies in the literature [54, 55]. However, little evidence is available to confirm the effect of cement thickness for retrievability. Leyla Sadighpour [56] evaluated the effect of two resin cements, PANAVIA and Unicem, on the retentive strength of zirconia. They found a statistically significant difference in the bond strength of cementation by PANAVIA and Unicem and concluded that types of resin cements, which cause differences in porosity, can influence the retention strength of zirconia restorations.

## 4. Clinical Trials

In terms of survival, clinical, and esthetical outcomes, a small number of clinical trial studies have assessed the overall success rate of zirconia crowns. In a systematic review, Jung et al. showed 4.5% chipping after an observation period up to 5 years [57]. In a retrospective study performed by Schwarz et al., it has revealed an incidence of chipping in zirconia crowns of 24.5% after almost 6 years [37]. In a study performed by Ortorp et al [3], it was reported that, according to the CDA evaluation, the clinical quality of the zirconia crowns was in the satisfactory rate, and patient satisfaction with these crowns was considerable. That study has also reported that there were no caries and adverse soft tissue reactions around the zirconia crowns. However, it was declared that a need for improved oral hygiene and scaling is significant. According to the results obtained by Ortorp et al., other clinical complications such as endodontic problems, loss of retention, and root fracture in zirconia crowns may be occurred; however, these complications had most likely other causes than the ceramic material.

In the study of Ortorp et al., the total incidence of complications was somewhat high; however, if the usual complications were excluded, for instance, excess of cement detected at next check-up, temporary pain after cementation, and loosened crowns that easily could be recemented, the complication rate was 9 percent. Before the zirconia era, the mean rate of complications incidence in a review of all-ceramic studies was 8 percent [58]. In the study of Ortorp et al., the rates of endodontic problems and loss of retention were higher (5 and 6, respectively) than the reported rates in the earlier all-ceramic studies (1% and 2%, respectively). In a systematic review [33], the 5-year survival rates were similar for the ceramic crowns and metal-ceramic crowns when used for anterior teeth but on average higher for the metal-ceramic crowns when used for posterior teeth (96% compared with for 93% ceramic crowns). In Ortorp et al.'s study, the 3-year survival rate for zirconia crowns was 93%. In another study, the 24-month survival rate for zirconia crowns was 90% [59]. It seems that it is hardly meaningful to make further detailed comparisons of the results of different studies which can be resulted from the differences in patient materials, outcome criteria, and study design. Encke et al. have reported that the mean fracture toughness for the ZrSiO4 ceramic is 5.16 MPa·m^1⁄2^ which is weaker than Zirconia-TZP (9.42 MPa·m^1⁄2^) but is comparable with In-Ceram Alumina (5.00 MPa·m^1⁄2^) [60, 61]. They also have shown that mean fracture strength for ZrSiO_4_ ceramic crowns, under moist conditions with 49 N in 1.2 million chewing cycles (5 years simulated) and temperature changed from 5 to 55°C, was 1789.6 N (±658.9). In a similar study performed by Okutan [62], the mean fracture loads for ZrSiO_4_ ceramic crowns in 1.2 million chewing cycles were consistent with rates obtained by Ortorp et al.'s study. However, they declared that cementation type may provide the different rates, for instance, mean fracture loads for ZrSiO_4_ ceramic crowns cemented with Ketac Cem was 1662 N (±433) and for crowns cemented with Panavia 21 EX was 1957 N (±806). The zirconia crowns have high chemical resistance and considerable biocompatibility. In Encke et al.'s study, the chemical solubility tested by the exposure method at 80°C in a 4% acetic acid solution is 7.2 *μ*g·cm^−2^.

## 5. Limitations of the Current Research

In this study, there are limitations that should be noted. The current study is a narrative review, and it has been criticised for lacking synthesis and rigour, although narrative reviews may be broader in scope than systematic reviews. In addition, narrative reviews have been criticised for rarely employing peer-reviewed methodologies, or duplicate curation of evidence, and for often failing to disclose study inclusion criteria. Despite these limitations, narrative reviews remain frequent within the literature, as they offer breadth of literature coverage and flexibility to deal with evolving knowledge and concepts.

## 6. Conclusion and Future Perspectives

In conclusion, the authors concluded that zirconia crowns are a promising prosthodontic valid alternative to metal ones and show good clinical performance based on observation and in vivo investigations. Although clinical long-term and follow-up studies are a vital requirement to conclude that zirconia has great reliability, it seems that zirconia crown restorations are both well tolerated and sufficiently resistant. However, several aspects remain to be studied and assessed. On top of all, the in vivo prospective studies on zirconia crowns to assessing the long-term clinical success; therefore, more research with long-term randomized clinical trials on the subject is currently suggested. In addition, an updated systematic review/meta-analysis, using PRISMA guidelines can be an appropriate way of reviewing the topic to provide clinicians with useful point of view in the decision-making process for use of these restorations.

## References

[1] Buser D., Sennerby L., De Bruyn H. (2017). Modern implant dentistry based on osseointegration: 50 years of progress, current trends and open questions. *Periodontol 2000*.

[2] Ceruso F., Barnaba P., Mazzoleni S. (2017). Implant-abutment connections on single crowns: a systematic review. *Oral & Implantology*.

[3] Örtorp A., Kihl M. L., Carlsson G. E. (2009). A 3-year retrospective and clinical follow-up study of zirconia single crowns performed in a private practice. *Journal of Dentistry*.

[4] Colombo M., Poggio C., Lasagna A., Chiesa M., Scribante A. (2019). Vickers micro-hardness of new restorative CAD/CAM dental materials: evaluation and comparison after exposure to acidic drink. *Materials*.

[5] Saab R. C., Da Cunha L. F., Gonzaga C. C., Mushashe A. M., Correr G. M. (2018). Micro-CT analysis of Y-TZP copings made by different CAD/CAM Systems: marginal and internal fit. *International Journal of Dentistry*.

[6] Lee Y., Chul Oh K., Kim N.-H., Moon H.-S. (2019). Evaluation of zirconia surfaces after strong-acid etching and its effects on the shear bond strength of dental resin cement. *International Journal of Dentistry*.

[7] Naveau A., Rignon-Bret C., Wulfman C. (2019). Zirconia abutments in the anterior region: a systematic review of mechanical and esthetic outcomes. *Journal of Prosthetic Dentistry*.

[8] Miyazaki T., Nakamura T., Matsumura H., Ban S., Kobayashi T. (2013). Current status of zirconia restoration. *Journal of Prosthetic Dentistry*.

[9] Denry I., Kelly J. R. (2008). State of the art of zirconia for dental applications. *Dental Materials*.

[10] Cakir Omur T., Gozneli R., Ozkan Y. (2019). Effects of silica coating by physical vapor deposition and repeated firing on the low temperature degradation and flexural strength of a zirconia ceramic. *Journal of Prosthodontics*.

[11] Guazzato M., Albakry M., Ringer S. P., Swain M. V. (2004). Strength, fracture toughness and microstructure of a selection of all-ceramic materials. part II. zirconia-based dental ceramics. *Dental Materials*.

[12] Andreiotelli M., Wenz H. J., Kohal R. J. (2009). Are ceramic implants a viable alternative to titanium implants? a systematic literature review. *Clinical Oral Implants Research*.

[13] Chevalier J., Loh J., Gremillard L., Meille S., Adolfson E. (2011). Low-temperature degradation in zirconia with a porous surface. *Acta Biomaterialia*.

[14] Guazzato M., Albakry M., Quach L., Swain M. V. (2004). Influence of grinding, sandblasting, polishing and heat treatment on the flexural strength of a glass-infiltrated alumina-reinforced dental ceramic. *Biomaterials*.

[15] Deville S., Chevalier J., Gremillard L. (2006). Influence of surface finish and residual stresses on the ageing sensitivity of biomedical grade zirconia. *Biomaterials*.

[16] Gehrke P., Dhom G., Brunner J., Wolf D., Degidi M., Piattelli A. (2006). Zirconium implant abutments: fracture strength and influence of cyclic loading on retaining-screw loosening. *Quintessence International*.

[17] Kohal R. J., Klaus G., Strub J. R. (2006). Zirconia implant supported all ceramic crowns withstand long term load: a pilot investigation. *Clinical Oral Implants Research*.

[18] Joo H.-S., Yang H.-S., Park S.-W. (2015). Influence of preparation depths on the fracture load of customized zirconia abutments with titanium insert. *Journal of Advanced Prosthodontics*.

[19] Silva N. R., Coelho P. G., Fernandes C. A., Navarro J. M., Dias R. A., Thompson V. P. (2009). Reliability of one piece ceramic implant. *Journal of Biomedical Materials Research Part B: Applied Biomaterials*.

[20] Kohal R. J., Finke H. C., Klaus G. (2009). Stability of prototype two piece zirconia and titanium implants after artificial aging: an in vitro pilot study. *Clinical Implant Dentistry and Related Research*.

[21] Kohal R. J., Wolkewitz M., Tsakona A. (2011). The effects of cyclic loading and preparation on the fracture strength of zirconium dioxide implants: an in vitro investigation. *Clinical Oral Implants Research*.

[22] Yildirim M., Fischer H., Marx R., Edelhoff D. (2003). In vivo fracture resistance of implant-supported all-ceramic restorations. *Journal of Prosthetic Dentistry*.

[23] Pecho O. E., Ghinea R., Ionescu A. M., Cardona J. C., Della Bona A., Del Mar Pérez M. (2015). Optical behavior of dental zirconia and dentin analyzed by Kubelka–Munk theory. *Dental Materials*.

[24] Heffernan M. J., Aquilino S. A., Diaz-Arnold A. M., Haselton D. R., Stanford C. M., Vargas M. A. (2002). Relative translucency of six all-ceramic systems. part I: core materials. *Journal of Prosthetic Dentistry*.

[25] Heffernan M. J., Aquilino S. A., Diaz-Arnold A. M., Haselton D. R., Stanford C. M., Vargas M. A. (2002). Relative translucency of six all-ceramic systems. part II: core and veneer materials. *Journal of Prosthetic Dentistry*.

[26] Cho M.-S., Yu B., Lee Y.-K. (2009). Opalescence of all-ceramic core and veneer materials. *Dental Materials*.

[27] Rimondini L., Cerroni L., Carrassi A., Torriceni P. (2002). Bacterial colonization of zirconia ceramic surfaces: an in vitro and in vivo study. *International Journal of Oral & Maxillofacial Implants*.

[28] Scarano A., Piattelli M., Caputi S., Favero G. A., Piattelli A. (2004). Bacterial adhesion on commercially pure titanium and zirconium oxide disks: an in vivo human study. *Journal of Periodontology*.

[29] Yeh S., Andreana S. (2004). Crown lengthening: basic principles, indications, techniques and clinical case reports. *New York State Dental Journal*.

[30] Manicone P. F., Iommetti P. R., Raffaelli L. (2007). An overview of zirconia ceramics: basic properties and clinical applications. *Journal of Dentistry*.

[31] Debis M. Z., Badr A. I., Agamy E., Mohamed G. F. (2019). Effect of using zirconia and metallic bar attachment overdentures on the supporting structures of mandibular edentulous ridge area (randomized control trial). *Indian Journal of Public Health Research and Development*.

[32] Rinke S., Lange K., Roediger M., Gersdorff N. (2015). Risk factors for technical and biological complications with zirconia single crowns. *Clinical Oral Investigations*.

[33] Pjetursson B. E., Sailer I., Zwahlen M., Hämmerle C. H. (2007). A systematic review of the survival and complication rates of all ceramic and metal–ceramic reconstructions after an observation period of at least 3 years. part I: single crowns. *Clinical Oral Implants Research*.

[34] Beier U. S., Kapferer I., Dumfahrt H. (2012). Clinical long-term evaluation and failure characteristics of 1,335 all-ceramic restorations. *International Journal of Prosthodontics*.

[35] Sailer I., Pjetursson B. E., Zwahlen M., Hämmerle C. H. (2007). A systematic review of the survival and complication rates of all ceramic and metal–ceramic reconstructions after an observation period of at least 3 years. part II: fixed dental prostheses. *Clinical Oral Implants Research*.

[36] Larsson C., Von Steyern P. V. (2010). Five-year follow-up of implant-supported Y-TZP and ZTA fixed dental prostheses. a randomized, prospective clinical trial comparing two different material systems. *International Journal of Prosthodontics*.

[37] Schwarz S., Schröder C., Hassel A., Bömicke W., Rammelsberg P. (2012). Survival and chipping of zirconia based and metal–ceramic implant supported single crowns. *Clinical Implant Dentistry and Related Research*.

[38] Moráguez O. D., Wiskott H. A., Scherrer S. S. (2015). Three-to nine-year survival estimates and fracture mechanisms of zirconia-and alumina-based restorations using standardized criteria to distinguish the severity of ceramic fractures. *Clinical Oral Investigations*.

[39] Güncü M. B., Cakan U., Muhtarogullari M., Canay S. (2015). Zirconia-based crowns up to 5 years in function: a retrospective clinical study and evaluation of prosthetic restorations and failures. *International Journal of Prosthodontics*.

[40] Beuer F., Schweiger J., Eichberger M., Kappert H. F., Gernet W., Edelhoff D. (2009). High-strength CAD/CAM-fabricated veneering material sintered to zirconia copings—a new fabrication mode for all-ceramic restorations. *Dental Materials*.

[41] Beuer F., Edelhoff D., Gernet W., Sorensen J. A. (2009). Three-year clinical prospective evaluation of zirconia-based posterior fixed dental prostheses (FDPs). *Clinical Oral Investigations*.

[42] Behr M., Rosentritt M., Loher H. (2008). Changes of cement properties caused by mixing errors: the therapeutic range of different cement types. *Dental Materials*.

[43] Son Y.-H., Han C.-H., Kim S. (2012). Influence of internal-gap width and cement type on the retentive force of zirconia copings in pullout testing. *Journal of Dentistry*.

[44] Derand T., Molin M., Kleven E., Haag P., Karlsson S. (2008). Bond strength of luting materials to ceramic crowns after different surface treatments. *European Journal of Prosthodontics and Restorative Dentistry*.

[45] Dérand P., Dérand T. (2000). Bond strength of luting cements to zirconium oxide ceramics. *International Journal of Prosthodontics*.

[46] Zhang Y., Lawn B. R., Rekow E. D., Thompson V. P. (2004). Effect of sandblasting on the long term performance of dental ceramics. *Journal of Biomedical Materials Research Part B: Applied Biomaterials*.

[47] Söderholm K.-J. M., Mondragon E., Garcea I. (2003). Use of zinc phosphate cement as a luting agent for Denzir™ copings: an in vitro study. *BMC Oral Health*.

[48] Cavalcanti A. N., Pilecki P., Foxton R. M. (2009). Evaluation of the surface roughness and morphologic features of Y-TZP ceramics after different surface treatments. *Photomedicine and Laser Surgery*.

[49] Wolfart M., Lehmann F., Wolfart S., Kern M. (2007). Durability of the resin bond strength to zirconia ceramic after using different surface conditioning methods. *Dental Materials*.

[50] Guazzato M., Quach L., Albakry M., Swain M. V. (2005). Influence of surface and heat treatments on the flexural strength of Y-TZP dental ceramic. *Journal of Dentistry*.

[51] Oblak C., Jevnikar P., Kosmac T., Funduk N., Marion L. (2004). Fracture resistance and reliability of new zirconia posts. *Journal of Prosthetic Dentistry*.

[52] Gultekin P., Gultekin B. A., Aydin M., Yalcin S. (2013). Cement selection for implant supported crowns fabricated with different luting space settings. *Journal of Prosthodontics*.

[53] Chee W. W., Duncan J., Afshar M., Moshaverinia A. (2013). Evaluation of the amount of excess cement around the margins of cement-retained dental implant restorations: the effect of the cement application method. *Journal of Prosthetic Dentistry*.

[54] Bernal G., Okamura M., Munoz C. A. (2003). The effects of abutment taper, length and cement type on resistance to dislodgement of cement retained, implant supported restorations. *Journal of Prosthodontics*.

[55] Bresciano M., Schierano G., Manzella C., Screti A., Bignardi C., Preti G. (2005). Retention of luting agents on implant abutments of different height and taper. *Clinical Oral Implants Research*.

[56] Sadighpour L., Geramipanah F., Fazel A., Allahdadi M., Kharazifard M. J. (2018). Effect of selected luting agents on the retention of CAD/CAM zirconia crowns under cyclic environmental pressure. *Journal of Dentistry (Tehran)*.

[57] Jung R. E., Pjetursson B. E., Glauser R., Zembic A., Zwahlen M., Lang N. P. (2008). A systematic review of the 5 year survival and complication rates of implant supported single crowns. *Clinical Oral Implants Research*.

[58] Goodacre C. J., Bernal G., Rungcharassaeng K., Kan J. Y. (2003). Clinical complications with implants and implant prostheses. *Journal of Prosthetic Dentistry*.

[59] Encke B., Heydecke G., Wolkewitz M., Strub J. (2009). Results of a prospective randomized controlled trial of posterior ZrSiO_4_ ceramic crowns. *Journal of Oral Rehabilitation*.

[60] Heydecke G., Butz F., Binder J., Strub J. (2007). Material characteristics of a novel shrinkage-free ZrSiO_4_ ceramic for the fabrication of posterior crowns. *Dental Materials*.

[61] Tinschert J., Natt G., Mohrbotter N., Spiekermann H., Schulze K. A. (2007). Lifetime of alumina‐and zirconia ceramics used for crown and bridge restorations. *Journal of Biomedical Materials Research Part B: Applied Biomaterials*.

[62] Okutan M., Heydecke G., Butz F., Strub J. (2006). Fracture load and marginal fit of shrinkage‐free ZrSiO_4_ all ceramic crowns after chewing simulation. *Journal of Oral Rehabilitation*.

